# Primary Mediastinal T‐Lymphoblastic Lymphoma With Airway Stenosis Diagnosed Using Endobronchial Ultrasound‐Guided Transbronchial Mediastinal Cryobiopsy: A Case Report

**DOI:** 10.1002/rcr2.70756

**Published:** 2026-09-07

**Authors:** Hiroaki Nagamine, Midori Bungo, Yuichiro Nagaki, Akira Todoriki, Kohei Niina, Koji Enomoto, Shigeki Kakuno, Toshiyuki Nakai, Kazuhiro Yamada, Tetsuya Watanabe, Kazuhisa Asai, Daiki Mukai, Joji Nagasaki, Hirohisa Nakamae, Tomoya Kawaguchi

**Affiliations:** ^1^ Department of Respiratory Medicine, Graduate School of Medicine Osaka Metropolitan University Osaka Japan; ^2^ Department of Hematology, Graduate School of Medicine Osaka Metropolitan University Osaka Japan

**Keywords:** cryobiopsy, endobronchial ultrasound‐guided cryobiopsy, mediastinal mass, T‐cell lymphoblastic lymphoma, transbronchial needle aspiration

## Abstract

Endobronchial ultrasound‐guided transbronchial needle aspiration (EBUS‐TBNA) is a minimally invasive technique for sampling mediastinal lesions but may be inadequate for diagnosing lymphoma because of limited tissue acquisition. We report a case of T‐lymphoblastic lymphoma (T‐LBL) successfully diagnosed using endobronchial ultrasound‐guided transbronchial mediastinal cryobiopsy (EBUS‐Cryo). An 18‐year‐old woman presented with an anterior mediastinal mass causing severe tracheal compression. EBUS‐TBNA followed by EBUS‐Cryo using a 1.1‐mm cryoprobe was performed under endotracheal intubation without complications. Although EBUS‐TBNA specimens were nondiagnostic because of crush artefacts and blood contamination, EBUS‐Cryo yielded well‐preserved tissue, enabling comprehensive immunohistochemical evaluation and a definitive diagnosis of T‐LBL. This case demonstrates that EBUS‐Cryo can provide sufficient tissue for diagnosing mediastinal T‐LBL and may be safely performed in carefully selected patients, even in the presence of substantial airway compression.

## Graphical abstract

EBUS‐Cryo provided larger, well‐preserved tissue specimens, enabling a definitive diagnosis of T‐lymphoblastic lymphoma when EBUS‐TBNA was nondiagnostic. EBUS‐Cryo was performed safely despite substantial tracheal compression, highlighting its potential as a minimally invasive diagnostic option for mediastinal lymphoma.

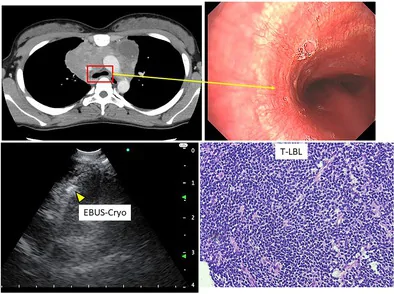

## 1 Introduction

Mediastinoscopy provides sufficient tissue for histopathological and immunophenotypic evaluation of mediastinal tumours but is highly invasive. In contrast, endobronchial ultrasound‐guided transbronchial needle aspiration (EBUS‐TBNA) is a minimally invasive procedure and has become widely used for the diagnosis of mediastinal lesions. However, its diagnostic yield for lymphoma remains limited because the small tissue volume and crush artefacts can impede accurate histopathological and immunohistochemical evaluation [1].

In recent years, cryobiopsy has gained attention as a useful technique for obtaining larger and better‐preserved tissue samples than those obtained by conventional forceps or needle aspiration biopsy. Previous studies have demonstrated its diagnostic utility in pulmonary lymphoma and lymphoproliferative disorders [2]. Furthermore, endobronchial ultrasound‐guided transbronchial mediastinal cryobiopsy (EBUS‐Cryo) has been introduced as a novel technique for sampling mediastinal lesions.

Here, we report a case of T‐lymphoblastic lymphoma (T‐LBL) presenting as a large anterior mediastinal mass with substantial tracheal compression, in which EBUS‐Cryo safely provided a larger and better‐preserved tissue sample, enabling a definitive diagnosis.

## 2 Case Report

**FIGURE 1:**
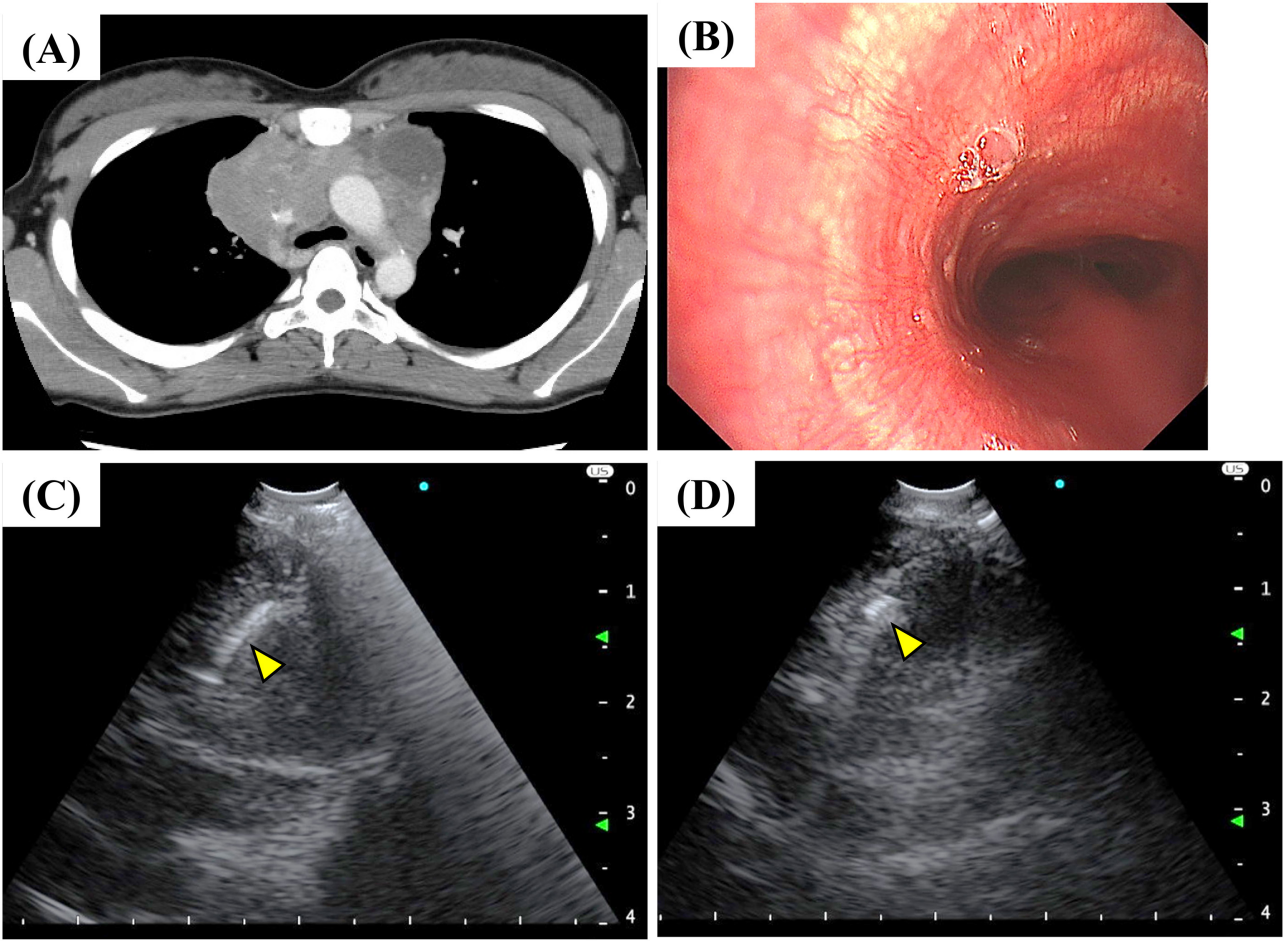
(A) Contrast‐enhanced chest computed tomography showing an anterior mediastinal mass compressing the trachea and carina. (B) Bronchoscopic findings demonstrate narrowing of the tracheal lumen due to extrinsic compression. (C) Endobronchial ultrasound (EBUS) images during EBUS‐transbronchial needle aspiration using a 22‐gauge needle (yellow triangle). (D) EBUS images during EBUS‐guided cryobiopsy using a 1.1‐mm cryoprobe (yellow triangle).

An 18‐year‐old woman was referred to our hospital for further evaluation of mediastinal widening, which was detected on a routine chest radiograph during a school health examination. She was asymptomatic, and the physical examination revealed no abnormal findings. Chest computed tomography demonstrated a large anterior mediastinal mass compressing the trachea and carina (Figure 1A).

**FIGURE 2:**
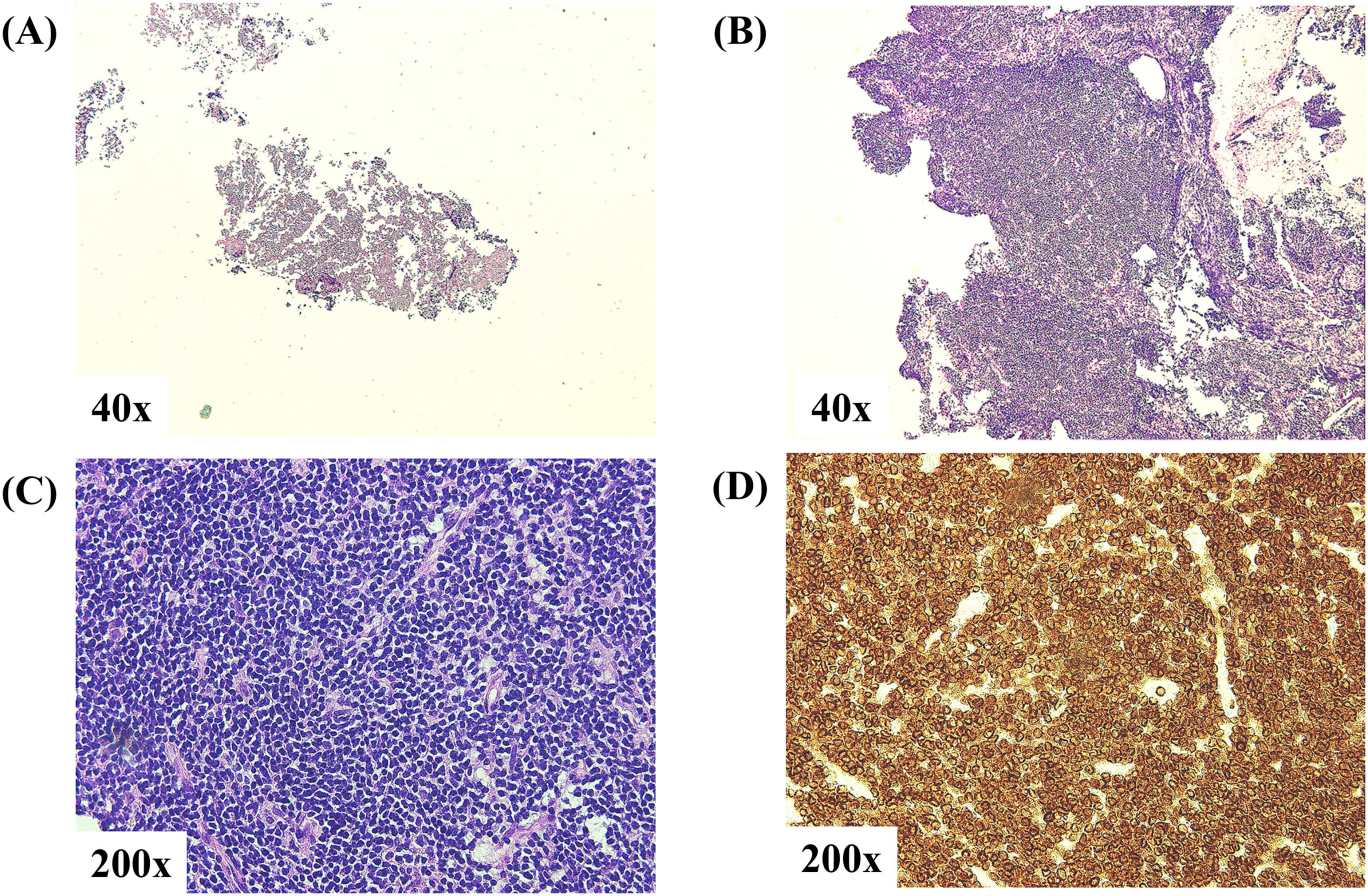
Pathological findings of the (A) endobronchial ultrasound‐guided transbronchial needle aspiration (EBUS‐TBNA) (40×) and (B, C) EBUS‐guided cryobiopsy (EBUS‐Cryo) specimen (b, 40×; c, 200×). EBUS‐Cryo provides larger and higher‐quality tissue specimens than EBUS‐TBNA. (D) Representative immunohistochemical staining shows positivity for CD3.

A bronchoscopy with planned EBUS‐TBNA and EBUS‐Cryo was performed to confirm the pathological diagnosis. Bronchoscopy revealed significant extrinsic compression of the distal trachea and carina, accompanied by marked narrowing of the lumen (Figure 1B). Under endotracheal intubation using an 8.5‐mm endotracheal tube, EBUS‐TBNA was performed three times at the same site using a 22‐gauge needle (ViziShot 2; Olympus, Tokyo, Japan) (Figure 1C). Subsequently, a 1.1‐mm cryoprobe (Erbe Elektromedizin GmbH, Tuebingen, Germany) was inserted through the tract created by the EBUS‐TBNA, and EBUS‐Cryo was performed (Figure 1D), resulting in the collection of a total of six cryobiopsy specimens. During EBUS‐Cryo, after a freezing time of 7 s, the cryoprobe was withdrawn en bloc together with the bronchoscope. Rapid on‐site cytologic evaluation was not performed. No procedure‐related adverse events occurred during or after the examination, and the patient was discharged the following day. Histopathological examination of the EBUS‐TBNA specimen failed to yield definitive findings due to tissue crush artefacts and blood contamination (Figure 2A). In contrast, the EBUS‐Cryo specimen showed well‐preserved tissue structure, revealing diffuse proliferation of atypical lymphocytes with round nuclei and a high nucleus‐to‐cytoplasm ratio (Figure 2B,C). These histopathological findings raised suspicion of malignant lymphoma, and immunohistochemical staining was performed for a definitive diagnosis. The atypical cells were positive for CD3 (Figure 2D), CD2, CD7, and CD99, supporting T‐cell lineage, while they were negative for CD20, CD4, CD5, CD8, CD1a, CD34, c‐kit, AE1/AE3, and SALL4. TdT expression was observed in only a small subset of cells. The absence of CD20 and AE1/AE3 expression argued against B‐cell lymphoma and epithelial malignancy, respectively, while the T‐cell–associated markers supported a T‐cell neoplasm. Although the limited TdT positivity was atypical for T‐LBL, the overall histopathological and immunophenotypic findings, together with the patient's young age and the location of the lesion, supported a final diagnosis of T‐LBL. Following diagnosis, she was referred to the Department of Haematology for initiation of systemic chemotherapy.

## 3 Discussion

In the present case, EBUS‐Cryo yielded a larger and better‐preserved tissue sample than EBUS‐TBNA, enabling a definitive diagnosis of T‐LBL. Moreover, the procedure was performed safely despite marked tracheal compression.

Accurate diagnosis of mediastinal tumours requires adequate tissue sampling for histopathological examination and extensive immunohistochemical analyses. Therefore, obtaining sufficient, well‐preserved tissue is essential for establishing a definitive diagnosis. EBUS‐TBNA has become a standard minimally invasive procedure for sampling mediastinal lesions and demonstrates high diagnostic accuracy for mediastinal lymph node metastases from lung cancer [3]. However, the amount of tissue obtained is often limited, and crush artefacts hinder histopathological and immunohistochemical evaluation. In the present case, these limitations prevented a definitive diagnosis by EBUS‐TBNA. In contrast, EBUS‐Cryo yielded tissue of sufficient quantity and quality for detailed immunohistochemical evaluation, enabling a definitive diagnosis of T‐LBL. This finding is consistent with previous reports suggesting that EBUS‐Cryo may complement EBUS‐TBNA when larger tissue samples are required, particularly in patients with suspected lymphoma or other benign lymphoproliferative disorders that require evaluation of tissue architecture and extensive immunophenotyping.

Safety is another important consideration when performing mediastinal cryobiopsy. Although EBUS‐Cryo is generally considered a relatively safe procedure, complications such as bleeding, pneumomediastinum, and mediastinal infection may occur [4, 5]. In patients with severe airway narrowing, such as the present case, even a small amount of bleeding may result in life‐threatening airway compromise. Cryobiopsy is generally associated with a risk of bleeding. However, the 1.1‐mm cryoprobe requires only a small puncture tract and may theoretically reduce the risk of procedure‐related complications compared with larger devices such as forceps and electrocautery devices. In the present case, despite marked tracheal and carinal compression, the procedure was completed without hypoxemia or airway compromise, and the patient was discharged the following day. These findings suggest that EBUS‐Cryo can be performed safely in carefully selected patients with substantial airway compression.

In conclusion, this case suggests that EBUS‐Cryo can provide adequate and better‐preserved tissue for the definitive diagnosis of mediastinal T‐LBL and may be safely performed in selected patients, even in the presence of substantial airway compression.

## Author Contributions

Hiroaki Nagamine contributed to the conceptualization, methodology, investigation, data curation, writing – original draft, writing – review and editing, visualization and project administration. Midori Bungo, Yuichiro Nagaki, Akira Todoriki, Kohei Niina, Koji Enomoto, Toshiyuki Nakai, Kazuhiro Yamada, Tetsuya Watanabe, Kazuhisa Asai, Daiki Mukai, Joji Nagasaki, Hirohisa Nakamae and Tomoya Kawaguchi contributed to the methodology and writing – review and editing. All authors reviewed and approved the final version of the manuscript.

## Consent

The authors declare that written informed consent was obtained for the publication of this manuscript and accompanying images and attest that the form used to obtain consent from the patient complies with the Journal requirements as outlined in the author guidelines.

## Conflicts of Interest

The authors declare no conflicts of interest.

## Data Availability

The data that support the findings of this study are available from the corresponding author upon reasonable request.
